# Peptidoglycan O-Acetylation as a Virulence Factor: Its Effect on Lysozyme in the Innate Immune System

**DOI:** 10.3390/antibiotics8030094

**Published:** 2019-07-18

**Authors:** Ashley S. Brott, Anthony J. Clarke

**Affiliations:** Department of Molecular & Cellular Biology, University of Guelph, Guelph, ON N1G 2W1, Canada

**Keywords:** peptidoglycan, O-acetylation, pathogenesis, lysozyme

## Abstract

The peptidoglycan sacculus of both Gram-positive and Gram-negative bacteria acts as a protective mesh and provides structural support around the entirety of the cell. The integrity of this structure is of utmost importance for cell viability and so naturally is the first target for attack by the host immune system during bacterial infection. Lysozyme, a muramidase and the first line of defense of the innate immune system, targets the peptidoglycan sacculus hydrolyzing the β-(1→4) linkage between repeating glycan units, causing lysis and the death of the invading bacterium. The O-acetylation of *N*-acetylmuramoyl residues within peptidoglycan precludes the productive binding of lysozyme, and in doing so renders it inactive. This modification has been shown to be an important virulence factor in pathogens such as *Staphylococcus aureus* and *Neisseria gonorrhoeae* and is currently being investigated as a novel target for anti-virulence therapies. This article reviews interactions made between peptidoglycan and the host immune system, specifically with respect to lysozyme, and how the O-acetylation of the peptidoglycan interrupts these interactions, leading to increased pathogenicity.

## 1. Introduction

Antimicrobial resistance is at the forefront of today’s medical crises. The World Health Organization (WHO) has acknowledged the severity of this issue, and in 2014 stated that a post-antibiotic era is a ‘very real possibility for the 21st century’ [1]. Both WHO and the Centers for Disease Control and Prevention (CDC) have published priority pathogen lists emphasizing key bacterial pathogens which are highlighted as being urgent or serious threats and for which there is a serious need for the research and development of novel antibiotics. Bacterial pathogens which were identified in both reports include drug-resistant strains of *Staphylococcus aureus, Streptococcus pneumoniae, Neisseria gonorrhoeae,* and *Campylobacter* species [2,3] (Table 1). With declining efficiencies of our current arsenal of clinically used antibiotics, it is imperative that new therapies are identified, or illnesses which were once curable may not continue to be so easily managed. As a whole, studies in the current resistance era focused on identifying novel antimicrobials have been met with limited success; it has been predicted that a new age of antibiotic discovery, the narrow-spectrum era, is on the horizon, but to reach this era, discovery strategies must be drastically altered to potentiate higher success [4]. New therapeutic strategies including combination drug therapy to exploit synergistic reactions or targeting bacterial systems which can work synergistically with the host immune system as a part of novel antivirulence strategies may prove advantageous. For this to occur, however, a greater understanding of how these pathogens evade immune detection, how unique virulence systems are employed, and how these alter host–microbe interactions must be acquired. 

The peptidoglycan (PG) sacculus represents a key structural component which is found uniquely within bacterial cell walls. PG forms an intricate mesh which encompasses the entirety of the bacterial cell and is responsible for maintaining cellular viability by resisting internal turgor pressure. The crucial role PG fulfills makes it a natural target for both antibiotics and the host immune system upon infection. Regarding the latter, lysozyme targets this macromolecular structure as a first line of defense against invading pathogens (recently reviewed in [6]). Many pathogens have developed a mechanism to render lysozyme ineffective, thus increasing their pathogenicity as well as the severity of infection. One such example is the O-acetylation of muramoyl residues within the PG sacculus, representing a substrate-level modification which directly inhibits lysozyme binding [7,8].

This review presents our current understanding of the role PG O-acetylation plays in pathogens to evade the host immune response and of which downstream complications often occur due to this important virulence factor. This information is discussed in the context of the development of new therapeutics and the potential use of PG O-acetylation systems as targets for novel anti-virulence strategies to combat pathogenic bacteria. 

## 2. Peptidoglycan Composition

**Figure 1:**
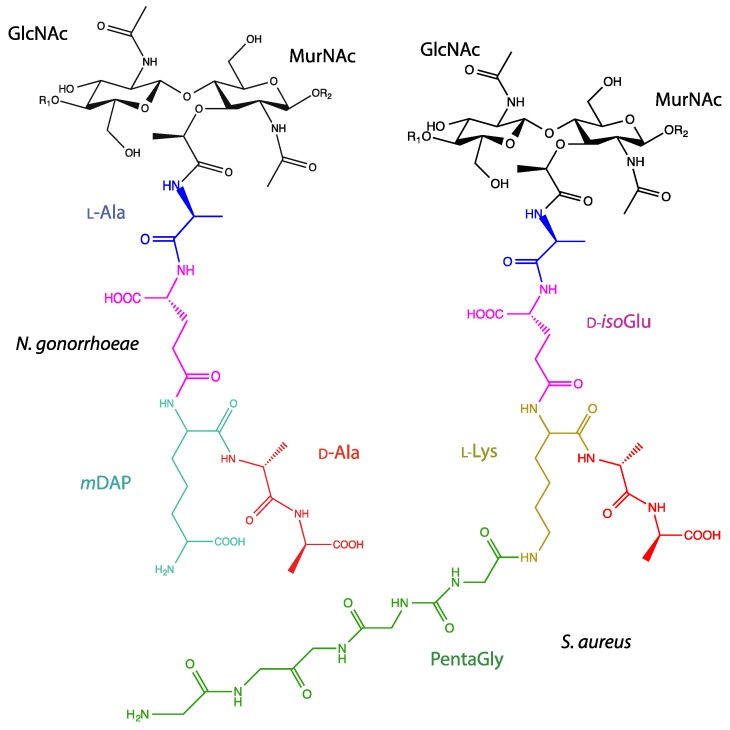
Structure of *N. gonorrhoeae* and *S. aureus* PG. The PG sacculus is composed of repeating units of GlcNAc-β(1→4)-MurNAc where the C-3 lactyl moiety of MurNAc is decorated with a stem peptide chain. The stem peptide varies the greatest between the two main classifications of bacteria. R_1_ and R_2_ denote the extending repeat units commencing with MurNAc or GlcNAc residues, respectively.

In both Gram-negative and Gram-positive bacteria, the PG sacculus represents a rigid stress baring layer located external to the cytoplasmic membrane. PG is composed of a conserved repeating disaccharide unit of β-(1→4) linked *N-*acetylglucosamine (GlcNAc) and *N*-acetylmuramic acid (MurNAc), with the exception of the terminal residue in Gram-negative bacteria and some Gram-positive bacteria, which is instead a non-reducing 1,6-anhydroMurNAc, containing an intramolecular ring between C-1 and C-6 (Figure 1). Glycan strand length has been found to be highly variable and does not appear to correlate with PG thickness; Gram-negative PG does not necessarily contain shorter glycan strand lengths than Gram-positive PG [9]. Average chain lengths exceeding 100 disaccharide units have been reported in species of *Bacillus*, while chain lengths of less than 10 disaccharide units have been found in strains of *Helicobacter pylori* [10,11]. It has also been found that average chain length can vary with strain and culture conditions, adding to the heterogenicity found within this large macromolecule [12].

The C-3 lactyl moiety of MurNAc residues are substituted with a peptide stem consisting of alternating l and d amino acids, a proportion of which is incorporated into covalent cross-linkages between MurNAc residues on neighboring strands (Figure 1). The nature of this crosslinking as well as the composition of the stem peptide defines the chemotype of the respective bacteria. Despite over 50 chemotypes being originally discovered, regions of the stem peptide are found to be conserved among most bacterial PG [13]. The first amino acid exists as a covalent modification on the C-3 lactyl moiety of MurNAc and is canonically l-Ala. This is followed usually by a d-Glu, although an amidated form, iso-d-Gln, has also been identified in a subset of bacteria [14]. The third amino acid position is the diamino acid, which is involved in the formation of covalent cross-linkages between neighboring peptide stems and is also the most variable between species. In Gram-negative bacteria, meso-diaminopimelic acid (*m*DAP) is often found in this position, while l-Lys is common amongst Gram-positive bacteria. Consecutive d-Ala residues constitute the final fourth and fifth positions, although in mature PG, the cleavage of both or solely the terminal d-Ala is often observed as a result of endogenous carboxypeptidases [15].

Greater variation exists within the cross-linkages between MurNAc residues, and this variation can be seen in both the position and composition of the interpeptide bridge. This covalent bond can be formed directly between two stem peptides via the amino acids at the third and fourth positions (*m*DAP/l-Lys to d-Ala), or between amino acids at the second position and fourth position (d-Glu to d-Ala), although the latter is mostly found within *Corynebacteria* and so is less frequently observed [13]. Interpeptide bonds can also be formed using an interpeptide bridge consisting of a single amino acid, as seen in *Bifidobacterium* with a single Gly residue, or several amino acids, as is seen in Staphylococcal species where bridges have been found to consist of up to 7 Gly residues or, as in the case of *Staphylococcus epidermidis*, Gly_2-4_ → l-Ser_1-2_ → Gly [14].

**Figure 2:**
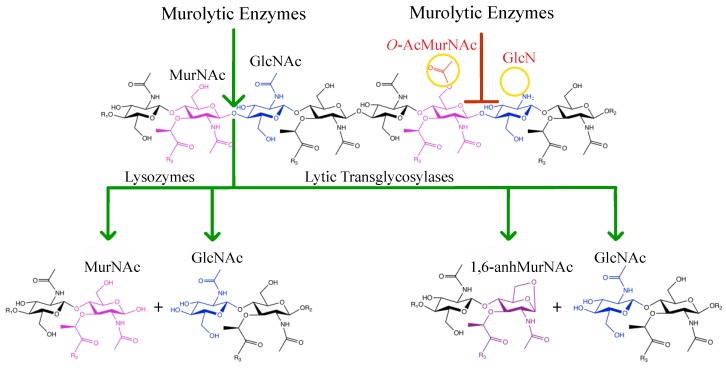
Lytic action and inhibition of murolytic enzymes. Lysozymes as well as endogenous lytic transglycosylases cleave the β(1→4) glycosidic linkage between MurNAc and GlcNAc. Lysozyme catalyzes a hydrolytic mechanism releasing two reaction products, one of which terminates in a reducing MurNAc moiety. LTs cleave via a non-hydrolytic mechanism, releasing an equivalent reaction product terminating with a 1,6-anhMurNAc residue. Both classes of enzymes are inhibited by MurNAc O-acetylation while the hydrolytic action of lysozymes is also inhibited by the N-deacetylation of GlcNAc.

In addition to the differences in stem peptide composition, a number of modifications may occur within the general PG structure (Figure 2). For example, the addition of a branching Gly residue on the α-carbon of d-Glu is found in species such as *Micrococcus* [16], or as very recently observed, on the l-ornithine of *Borrelia burgdorferi* stem peptides (ornithine replaces *m*DAP in this bacterium) [17]. More commonly found, however, are modifications to the glycans, which include N-deacetylation, N-glycosylation and O-acetylation (recently reviewed in [5,18]). Both N-deacetylation and O-acetylation are known to affect key interactions between invading bacteria and the host immune system. The latter is the focus of this review and will be discussed in detail in subsequent sections. 

## 3. Peptidoglycan and Host Immune Interactions

The necessary level of integrity that must be upheld within the PG sacculus to maintain a viable cell leaves a natural “Achilles heel” within invading pathogens. Host immune systems across the animal kingdom have evolved to take advantage of this as the PG sacculus is often one of the first targets during an immune attack. Lysozyme, a cationic muramidase, is often referred to as the first line of defense of the innate immune system. It is one of the most abundant cationic antimicrobial peptides within the body as it is found in high concentrations, up to 1 mg/mL within secretions such as saliva, tears, urine, at mucosal surfaces and within the airway, as well as in the blood, liver and phagocytes [19,20]. Although the cationic properties of lysozyme have been found to possess antimicrobial activity, as it is able to insert and form pores within negatively charged bacterial membranes [21], the main bacteriolytic effect is exerted by its ability to act as a PG hydrolase [22]. This activity is effective against both Gram-positive and Gram-negative bacteria, despite the latter containing an additional barrier in the form of the outer membrane as the activity of lactoferrin and defensins of the immune system, in addition to the cationic properties of lysozyme, aid in permeabilizing the outer membrane and exposing the PG sacculus [23,24]. Other PG–immune system interactions include the use of PG subunits released from the existing sacculus by either the activity of lysozyme or the natural turnover of PG to act as signals of a bacterial infection, leading to increased immune activation. There is a considerable amount of information regarding the interactions of PG and its metabolites with the immune system, and the reader is directed to several recent comprehensive reviews on the subject [5,25,26]. The following summarizes those interactions that directly involve lysozyme.

**Figure 3:**
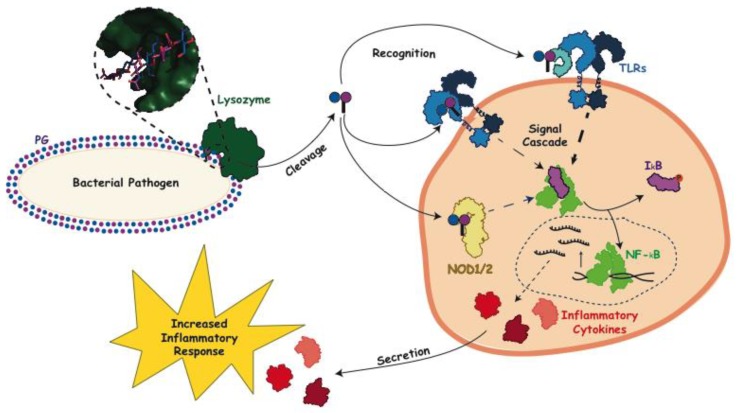
General immune inflammatory response following the lytic action of lysozyme. Upon bacterial invasion lysozyme (PDB ID: 5NJQ, dark green) acts to cleave the PG sacculus and release monomers consisting of GlcNAc-MurNAc (peptide). These monomers then act as a signaling molecule binding to extracellular pattern recognition receptors on the surface of immune cells, e.g., toll-like receptor 2 (TLR2) (PDB ID: 1FYW, 3A7C, blue), and initiate a response which is aided by CD14 (PDB ID: 1WWL, teal). PG signaling monomers can also bind intracellular nucleotide-binding oligomerization domain-containing (NOD) proteins (model of human NOD1 generated using PHYRE2 [33] using PDB ID 6B5B as a template, yellow) which also generates a signal to initiate an inflammatory response. Cytoplasmic NF-*_k_*B is inhibited by I*_k_*B (PDB IDs: 1NFI, 1VFI, light green and purple, respectively). The activation of NF-*_k_*B is initiated by the release the I*_k_*B (due to the phosphorylation of the later) and its translocation of to the nucleus where it functions as a transcription factor for the production of inflammatory cytokines (red), such as TNFα, IL-1β and IL-6 (PDB IDs: 1TNF, 1I1B, and 1IL6, respectively), which are then secreted from the cell to increase the inflammatory response.

PG is synthesized by almost all species of bacteria, but as a heteropolymer of both carbohydrates and peptides, a related glycan is not produced by eukaryotic organisms making it an efficient signal of pathogenic bacterial infections. Lysozyme liberates PG subunits, allowing them to freely circulate within the host, a signal which can be received even before lysozyme has achieved bacterial lysis [27]. Pattern recognition receptors downstream of lysozyme degradation recognize this signal and initiate the production of pro-inflammatory cytokines, as was seen in the case of *S. aureus* infection, where toll-like receptor 2 (TLR2) was found to be a necessary extracellular receptor (Figure 3) [28]. CD14, a glycosylphosphatidylinositol (GPI) protein found on the surface of macrophages, has also been shown to interact with PG fragments and enhance TLR2 activity in the case of both *S. aureus* and *Bacillus subtilis.* This interaction activates nuclear factor kappa-light-chain-enhancer of activated B cells (NF-*_K_*B), a ubiquitous family of transcription factors, and initiates its translocation to the nucleus [29]. NF-*_K_*B is also activated through a signal cascade in phagocytes via the recognition by the intracellular receptors nucleotide-binding oligomerization domain-containing protein 1 and 2 (NOD1 and NOD2). Gram-negative, and some Gram-positive, PG fragments containing γ-d-Glu-*m*DAP activate NOD1, while it has been demonstrated that for NOD2, the minimal fragment needed for recognition is a MurNAc residue, suggesting that it could function as a universal PG receptor [30,31]. It should be noted, however, that although lysozyme-released fragments adequately stimulate TLR2 and NOD2, released monomers containing 1,6-anhMurNAc products have been shown to have much weaker activation abilities [32]. Nonetheless, in all cases, NF-*_K_*B activation increases the immune response by increasing the expression of proinflammatory cytokines, such as tumor necrosis factor (TNF)α, interleukin (IL)-1β and IL-6, completing the cycle by which PG degradation via lysozyme lytic activity indirectly strengthens the immune response [5].

## 4. Interaction of Lysozyme with PG

The structure of hen egg-white lysozyme (HEWL) was the first enzyme to be modelled by X-ray crystallography [34], and a great amount of detail has since been determined with respect to its catalytic mechanism of action. As a muramidase, HEWL cleaves the β(1→4) glycosidic linkage between MurNAc and GlcNAc residues within PG. These residues are positioned at subsites −1 and +1, respectively, of the extended active site of this enzyme, which is located in a deep groove between two major protein domains. A total of six subsites (−4 to +2, named A to F) accommodate three disaccharide repeat units and make extensive contacts with each [35,36]. These interactions include hydrogen bonds between main-chain CO and NH groups comprising subsites A, C, and E and both the NH and the carbonyl oxygens of the acetamido groups of three GlcNAc residues. PG binding is also stabilized by a series of hydrophobic interactions and hydrogen bonds formed between the main chain of residues present in subsites B, D, and F and the C-6 OH of MurNAc. This network of interactions is fundamental for productive binding and their disruption by the de-N*-*acetylation of GlcNAc and/or the O-acetylation of MurNAc residues inhibits lysozyme activity. Many pathogenic species of bacteria that are able to evade this lytic activity of lysozyme do so using the latter resistance mechanism, viz. the acetylation of the C-6 OH of MurNAc (Figure 2), a feature first recognized over 60 years ago [37].

## 5. O-Acetylation of Peptidoglycan

The acetylation of the C-6 hydroxyl of MurNAc, here referred to as O-acetylation, is a common modification found across many bacterial species, particularly within pathogens; notable exceptions include *Escherichia coli*, some *Pseudomonads* (including *P. aeruginosa*), and *Bacillus anthracis* (Table 1) [38,39,40]. The extent of the modification in producing cells varies with species, strain, and even culture conditions. These differences have been used to demonstrate the dependence of lysozyme resistance on the degree of PG O-acetylation. As the C-6 hydroxyl groups of the *N*-acetylmuramoyl residues bound in subsites B, D and F of lysozyme make contact with amino acid residues forming the binding cleft, each interaction would be sterically hindered by the presence of *O*-acetyl groups and thus weaken binding affinity. A kinetic analysis of the hydrolysis of PG samples isolated from 14 different *Proteus mirabilis* strains with increasing *O-*acetyl content by both HEWL and human lysozyme content demonstrated that the overall change in the standard Gibbs free energy of activation (Δ(ΔG)) of the enzymes increased with increasing O-acetylation [41]. Similarly, a direct correlation was observed between the degree of O-acetylation of gonococcal PG and the extent of its solubilization by these two lysozymes [42,43,44,45]. With these findings, PG O-acetylation has been recognized as a key virulence factor for infection [7]. Such was clearly demonstrated for Gram-positive bacteria in a study by Bera et al. [46] who analyzed a collection staphylococcal species for both lysozyme resistance and O-acetylation. Two clear groups emerged from this analysis, as it was seen that species known to be pathogenic were clustered into one group, which were both lysozyme sensitive as well as produced *O*-Ac-MurNAc, and non-pathogenic species were clustered into a second group defined by lysozyme sensitivity and a lack of O-acetylation.

Despite the clear role that O-acetylation plays in pathogenicity, the development of knowledge in the field has occurred at a modest pace. In the 1970s and 1980s, O-acetylation was identified as a maturation event following PG biosynthesis. Through the use of pulse-chase experiments, O-acetylation in several species identified a temporal relationship between O-acetylation and the incorporation of the lipid-linked PG precursor Lipid II into the existing sacculus. Investigators found that O-acetylation only occurred after the initial burst of transpeptidation activity and did not appear to precede the synthesis of the mature sacculus [47,48]. This complemented additional studies which demonstrated that O-acetyl content increased after transpeptidation and that the use of penicillin G to inhibit transpeptidation activity resulted in a decrease in levels of *O*-acetyl PG [49,50,51,52]. 

**Figure 4:**
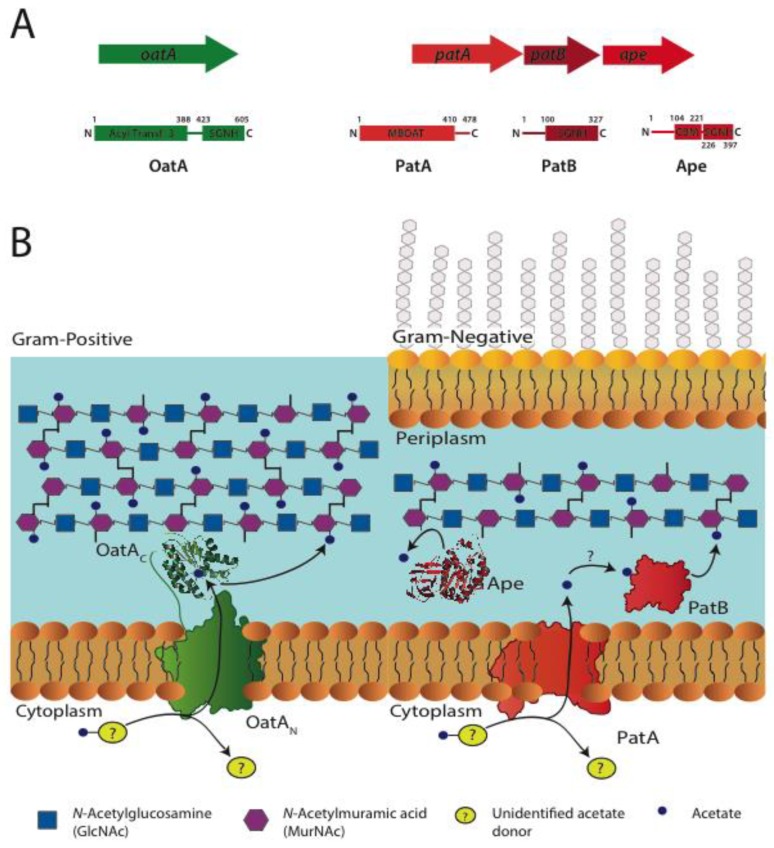
Gene organization and proposed models for PG O-acetylation. (**A**) Organization of genes encoding enzymes involved in the O-acetylation and de-O-acetylation of PG, and the domain organization of respective protein products. (**B**) Current models of O-acetylation systems for Gram-positive and Gram-negative bacteria. In both cases, a membrane spanning *O*-acetyltransferase (OatA_N_/PatA) acquires an acetyl group from an unidentified cytoplasmic source for its translocation to the external leaflet of the cytoplasmic membrane. The acetyl group is received by the dedicated PG *O*-acetyltransferase (OatA_C_/PatB) which modifies the C-6 OH of MurNAc. In Gram-negative bacteria, an esterase, Ape, is produced to remove the modification allowing for remodeling of the sacculus. Figure adapted from [5].

The genes encoding the Gram-negative O-acetylation system were not identified until 2005 [53] with the release of the *N. gonorrhoeae* genome. The sequence of *P. aeruginosa* AlgI, a membrane bound *O*-acyltransferase (MBOAT) involved in the O-acetylation of alginate [54], was used to identify the sequence of a potential O-acetyltransferase within the *N. gonorrhoeae* genome. An AlgI paralog was found within a three-gene operon, named OAP (*O*-acetyl PG), the gene products of which were proposed to be involved in the O-acetylation of PG given the absence of alginate polysaccharide synthesis in *N. gonorrhoeae* [53]. The hypothetical protein encoded by the first gene of this cluster-like AlgI was identified as an MBOAT and named PG *O-*acetyltransferase A (PatA). Its participation in the O-acetylation of PG was demonstrated by a series of in vivo complementation experiments with *N. gonorrhoeae* [55]. Found directly downstream from *patA,* a gene encoding a protein identified as being a member of the SGNH/GDSL hydrolase family of esterases was predicted to be an *O*-acetyl-PG esterase and named Ape2 [53]. Ape2 was later characterized biochemically and found, in fact, to be a second peptidoglycan *O*-acetyltransferase and thus renamed PatB [56]. The third and final gene product in the OAP operon, Ape1, also a member of the SGNH/GDSL hydrolase family, was confirmed to function as an authentic *O*-acetyl-PG esterase (Figure 4A) [57]. 

As a putative integral membrane protein, PatA is hypothesized to act as a shuttle to transport acetyl groups from a cytoplasmic source, likely acetyl-CoA, across the inner membrane for their presentation to PatB which is localized to the periplasm [56]. Being the dedicated *O*-acetyl PG transferase, PatB would then use this acetyl group to directly modify the C-6-hydroxyl of MurNAc residues already existing within the PG sacculus (Figure 4B) [56]. The nature of the transfer of acetyl groups between PatA and PatB remains unknown. It is possible that acetylated PatA serves as the direct acetyl donor for PatB through a protein–protein interaction. Alternatively, and perhaps more likely, an acetylated intermediate is formed involving a small carrier molecule available at the periplasmic face of the cytoplasmic membrane.

Also in 2005, the gene *oatA* (named for *O-*acetyltransferase A) was found to be responsible for the O-acetylation of PG in *S. aureus,* and in *S. pneumoniae* the following year [58,59]. OatA is a bimodular protein consisting of a putative N-terminal, integral membrane domain (OatA_N_) linked to a C-terminal extracellular domain (OatA_C_) [60]. The two domains of OatA are predicted to fulfill similar functions as the two-component Gram-negative system. Thus, despite being a member of the Acyltransferase-3 family and not an MBOAT like PatA, OatA_N_ would serve to shuttle acetyl moieties from a cytoplasmic donor, which is also predicted to be Ac-CoA, across the membrane. Once on the extracellular surface, this acetyl group would be presented to the catalytic transferase OatA_C_ domain, which is also a member of the SGNH/GDSL hydrolase like PatB, for the acetylation of PG [60]. To date, PatB from *N. gonorrhoeae* and OatA_C_ from both *S. aureus* and *S. pneumoniae* have been biochemically characterized and the structure–function relationships of these enzymes have been assessed [61,62,63,64]. For a detailed summary of the reaction mechanisms and structural characterizations identified to date, the reader is referred to a recent review on the mechanism of O-acetylation in both Gram-negative and Gram-positive bacteria [5].

## 6. Physiological and Pathobiological Significance of PG O-Acetylation

### 6.1. Physiological Role

Aside from contributing to pathogenicity and infection, PG O-acetylation plays a significant physiological role within the producing cell, at least with Gram-negative bacteria. This notion is supported by the fact that no strain of a species that normally O-acetylates its PG has been found to be devoid of the modification, even if it has to compensate for its loss of PatA/PatB activity. For example, *N. gonorrhoeae* RD5 contains a 26-base pair deletion mutation within its *patA* gene which results in a truncated, non-functional, PatA [54]. Nonetheless, its PG O-acetylation levels are between 10 and 15% with respect to MurNAc content, whereas wild-type strains ordinarily contain 40–60% O-acetylation [42,43,44,45]. The reason for maintaining a basal amount of O-acetylation is the intracellular regulatory role it plays in PG biosynthesis and maturation. 

The metabolism of PG in Gram-negative bacteria requires the activity of PG lytic enzymes, autolysins, to provide new sites for biosynthesis and create pores for the insertion of wall-spanning protein complexes, such as flagella and secretion systems (reviewed in [15]). The major glycolytic enzymes produced by Gram-negative bacteria for this purpose are the lytic transglycosylases (LTs) [65]. As with lysozyme, LTs cleave the β(1→4) glycosidic linkage between MurNAc and GlcNAc. However, they catalyze this lysis via a non-hydrolytic reaction mechanism which results in the formation of a 1,6-anhMurNAc product [66] as opposed to the reducing MurNAc released by lysozyme (Figure 2). As their intramolecular reaction product requires a free C-6 hydroxyl group on the MurNAc residue forming the scissile bond to be cleaved, its O-acetylation totally precludes LT activity. Thus, PG O-acetylation provides a control mechanism of LT activity at the substrate level [65,67,68]. Indeed, with its reduced level of PG O-acetylation, *N. gonorrhoeae* RD5 is a highly autolytic strain and it has the highest PG turnover rates of all *N. gonorrhoeae* strains analyzed to date [45,55]. It follows that RD5 is also highly sensitive to the activity of lysozyme when the enzyme is able to access its PG substrate [55].

As noted above, bacteria producing PatA and PatB for PG O-acetylation also produce Ape, an essential esterase that functions in the periplasm to remove the blocking *O-*acetyl group and thereby permit LT activity when required for PG metabolism [57]. Nothing is known about how Ape is regulated, but it would not be surprising to find that it associates with the LTs that comprise the multi-protein complexes known as the divisosome and/or elongasome [69,70]. These complexes include, in addition to LTs, specific penicillin-binding proteins, the transglycosylases and transpeptidases responsible for PG biosynthesis [71] related to cell division and cylindrical wall growth, respectively. Ape may also associate with other LTs that are used to provide pores for the assembly of cell-wall spanning structures.

Rather than LTs, the major glycolytic autolysins of Gram-positive bacteria are β-*N*-acetylglucosamindases, hydrolases that cleave between GlcNAc and MurNAc residues of PG [14]. These enzymes are not inhibited by the O-acetylation of MurNAc and so these bacteria do not require an Ape homolog for general PG metabolism. Thus, the O-acetylation of PG in these bacteria would appear to be mainly a defensive measure against host lysozyme attack. Intriguingly, whereas the modification does not seem to play a regulatory role in PG metabolism, the non-catalytic presence of the PG *O-*acetyltransferase OatA may play a key role in the spatio-temporal control of cell septation, at least with *Lactobacillus plantarum* [72].

### 6.2. Pathobiology of PG O-Acetylation

The pathobiological consequences caused by the resistance of O-acetylated PG to lysozyme hydrolysis beyond cell killing have long been known. Studies in the 1980s demonstrated that large-molecular-weight fragments of PG persist in the circulatory systems of hosts to induce a variety of effects, including complement activation, pyrogenicity, somnogenicity, and arthritogenicity (reviewed in [73]). The direct relationship between the induction of these effects by PG and its O-acetylation was clearly demonstrated in a series of animal model studies involving strains of *N. gonorrhoeae* and *S. aureus* that varied in their levels of this modification (reviewed in [74]). Subsequent studies by the Rosenthal group demonstrated the suppression of food consumption and body weight gain in rats following intraperitoneal injections of O-acetylated gonorrheal PG [75,76]. More recently, the role that PG O-acetylation plays in the development of septic arthritis by *S. aureus* was clearly demonstrated [77]. In this latter study, wild-type *S. aureus* SA113 and a Δ*oatA* mutant were used separately to inoculate mice, and the two bacterial strains were assessed for their ability to persist in the host, as well as the level of disease progression they induced. Confirming an earlier study [78], the non-O-acetylated PG was found to induce a milder form of the disease compared to mice inoculated with the wild-type strain as they had significantly lower levels of both joint destruction and a decreased bacterial load [77]. 

Further studies involving Δ*oatA* strains of *S. aureus* have shown that O-acetylated PG helps the pathogen evade immune detection and activation of the inflammasome [79]. Wild-type cells, but not those of a Δ*oatA* strain, were observed to inhibit the production of cytokine IL-1β, while their O-acetylated PG concomitantly rendered them resistant to killing by lysozyme within macrophages, thus increasing their potential for survival and pathogenicity. Furthermore, the O-acetylated PG from wild-type *S. aureus* appears to decrease the efficiency of helper T-cell priming, thereby permitting reinfection by this pathogen [80]. Similar studies demonstrating both escape from the immune response and survival within macrophages resulting from the O-acetylation of PG have been made with other Gram-positive (*Streptococcus iniae* [81], *Listeria monocytogenes* [82]) and Gram-negative human pathogens (*Helicobacter pylori* [83], and *Neisseria meningitidis* [84]). It is interesting to note that the presence of O-acetylation does not appear to have a direct effect on the recognition of PG by NOD2. This suggests that any weakening of NOD2 activation is a result of the inhibition of the lysozyme lytic activity that is required to generate the small-molecular-weight NOD2 ligands [27].

## 7. Discussion: Targeting O-Acetylation as a Novel Anti-Virulence Target

With the continuing emergence of antibiotic resistant strains, the identification of novel antimicrobial targets and the development of new antibiotics is more critical than ever before. However, this is a task which must be met with new, cutting-edge strategies should we want to truly work towards identifying long-lasting solutions and not simply prolong what seems to be an inevitable post-antibiotic era. Many of our most historically successful antibiotics—for example, β-lactams (penicillins, cephalosporins) and glycopeptides (vancomycin)—target PG biosynthesis and maturation events, causing the lysis of the bacteria [85]. Although the bactericidal nature of these antibiotics is what makes them so efficient in clearing bacterial infections, it is also their weakness. Lysis of the cell is a fatal conclusion and is not desirable for any organism. Therefore, it should be no surprise that this selective pressure to remain viable has led bacteria to evolve and combat these antimicrobials with their own unique resistance mechanisms. We have entered an arms race whereby the rate at which bacteria can evolve is outpacing our ability to identify new resistance mechanisms as they emerge and combat them. As a result, there has been an increase in the number of cases of bacterial infections from multidrug resistant strains worldwide, creating the “antibiotic crisis” [86]. 

More recent efforts to combat bacterial infections have focused on identifying novel anti-virulence strategies. These strategies aim to weaken the bacterium’s pathogenicity without weakening the overall fitness of the bacterium, to the point that the antibiotic is seen as a large enough threat to warrant a resistance strategy. This is done by targeting virulence factors; for example, adhesion to inhibit host colonization, or O-acetylation (reviewed in [86,87]). The potential of PG O-acetylation as an attractive and viable anti-virulence target is underscored by results of a number of animal studies involving mutant strains of pathogens with reduced PG O-acetylation levels. A direct correlation was observed between reduced pathogenicity and enhanced susceptibility of PG to lysozyme with mutants of *S. aureus* [46,58], *S. pneumoniae* [81], *S. iniae* [88], *Streptococcus suis* [89], *Enterococcus faecalis* [90,91], *L. monocytogenes* [82,92], *H. pylori* [83], and *N. meningitidis* [84].

Targeting PG O-acetylation for antibiotic development has a number of attractive features. In addition to rendering the invading bacterium more susceptible to lysozyme, treated infections would be cleared with greater efficiency by the enzyme, thereby minimizing, if not eliminating, downstream medical complications such as arthrogenicity. Also, the highly conserved glycan component of the PG may prove to be a longer sustaining target. Most antibiotics that target PG metabolism function to inhibit reactions involving the stem peptide which, compared to the glycan backbone, are highly variable and can be modified with little cost to the host bacterium. That O-acetylation does not appear to have a housekeeping function in the metabolism of PG in Gram-positive pathogens minimizes any selective pressure for its retention and increased resistance. Indeed, engineered Δ*oatA* strains appear to grow at rates comparable to wild-type strains when cultured in the absence of lysozyme [58,59,73,78,79,80,88,89]. The issue with Gram-negative bacteria is different given the physiological role O-acetylation plays in controlling the LTs. It is clear that there is a requirement to retain a minimal level of PG O-acetylation for cells to survive. This is evident from the existence of *N. gonorrhoeae* RD5, where cells appear to compensate for their lack of a functional PatA to provide a basal level of O-acetylation. Presumably, another acetyltransolocase is recruited for this purpose. This being said, an inhibitor of PG O-acetylation would likely target all enzymes involved to not only make cells more susceptible to lysozyme but also interfere with PG metabolism through the deregulation of LT activity. This combined effect may prove too broad to overcome single mutations, thereby reducing rates of resistance. Another attractive feature of targeting PG O-acetylation is that the human microbiota is composed primarily of non-O-acetylating bacteria. Consequently, therapeutic regimes would not greatly affect the normal function of these bacteria, and the possible derivation of any resistance mechanisms from them would be minimized, if not precluded. 

## 8. Conclusions

In order to stand our ground in the fight against antibiotic resistance, novel strategies must be developed. Targeting PG O-acetylation as an anti-virulence strategy represents a new avenue which is now being explored and has the potential to be a longer lasting solution. Recently, we demonstrated the possibility that inhibitors of PatB and OatA with antibacterial activity can be isolated [87] and thus provided a preliminary proof of principle that is worthy of further investigation. 

## Figures and Tables

**Table 1:** Bacterial species highlighted in the World Health Organization (WHO) and Center for Disease Control and Prevention (CDC) priority pathogen reports^1^ that produce O-acetylated peptidoglycan (PG)^2^.

Gram-positive	Gram-negative
*Clostridium difficile*	*Enterobacteriaceae* ^3^
*Enterococcus * (*faecium*)	*Neisseria gonorrhoeae*
*Staphylococcus aureus* (incl. MRSA/VRSA)	*Acinetobacter*
*Mycobacterium tuberculosis*	*Helicobacter pylori*
*Streptococcus pneumoniae*	*Campylobacter* spp.
Group A/B *Streptococcus*	*Shigella* spp.

^1^ Listed in descending order of risk factor according to the reports. ^2^ Known (red) or predicted (black) to produce O-acetylated PG based on genomic analysis [5]. ^3^ Identified in select species within the order, but not *E. coli.*
